# Supplementary material for: The Effect of Herbal Medicine on Suicidal Behavior: A Protocol for Systematic Review and Meta-Analysis
Source: Healthcare (Basel). 2023 May 11;11(10):1387. doi: 10.3390/healthcare11101387 (PMC10218662; doi:10.3390/healthcare11101387)
Supplement: Supplementary file 1 [file healthcare-11-01387-s001.zip › Table S2.pdf]

Table S2. Search strategy to be used in each database.

Database	Search strategy
MEDLINE via PubMed	#1. Suicide [Mesh Terms] OR “Self Mutilation” [Mesh Terms] OR self-harm OR self-poisoning OR self-injur* OR “Self Mutilation” OR “attempted suicide” OR suicid*<br><br>#2. “Drugs, Chinese Herbal” [Mesh Terms] OR “Medicine, Chinese Traditional” [Mesh Terms] OR “Medicine, Kampo” [Mesh Terms] OR “Medicine, Korean Traditional” [Mesh Terms] OR “Korean medicine” OR “Chinese medicine” OR “Oriental medicine” OR “Kampo medicine” OR “herbal medicine” OR decoction*<br><br>#3. #1 AND #2
Excerpta Medica Databasevia Elsevier	#1. ‘suicidal behavior’/exp OR ‘suicide’/exp OR ‘automutilation’/exp OR self-harm OR self-poisoning OR self-injur* OR “Self Mutilation” OR “attempted suicide” OR suicid*<br><br>#2. ‘Chinese medicine’/exp OR ‘Chinese medicine’ OR ‘Kampo medicine’/exp OR ‘Kampo medicine’ OR ‘Korean medicine’/exp OR ‘Korean medicine’ OR ‘herbal medicine’ OR ‘Oriental medicine’/exp OR ‘Oriental medicine’ OR ‘decoction*’<br><br>#3. #1 AND #2
Cochrane Central Register of Controlled Trials	#1. MeSH descriptor: [Suicide] explode all trees<br><br>#2. MeSH descriptor: [Self Mutilation] explode all trees<br><br>#3. self-harm OR self-poisoning OR self-injur* OR “Self Mutilation” OR “attempted suicide” OR suicid*<br><br>#4. MeSH descriptor: [Drugs, Chinese Herbal] explode all trees<br><br>#5. MeSH descriptor: [Medicine, Chinese Traditional] explode all trees<br><br>#6. MeSH descriptor: [Medicine, Kampo] explode all trees<br><br>#7. Korean medicine OR Chinese medicine OR Oriental medicine OR Kampo medicine OR herbal medicine OR decoction*<br><br>#8. (#1 OR #2 OR #3) AND (#4 OR #5 OR #6 OR #7) in Trials
Cumulative Index to Nursing and Allied Health Literature via EBSCO	#1. SU Suicide OR SU Self Mutilation OR TX self-harm OR TX self-poisoning OR TX self-injur* OR TX Self Mutilation OR TX attempted suicide OR TX suicid*<br><br>#2. SU Drugs, Chinese Herbal OR SU Medicine, Chinese Traditional OR SU Medicine, Kampo OR SU Medicine, Korean Traditional OR

	TX Korean medicine OR TX Chinese medicine OR TX Oriental medicine OR TX Kampo medicine OR TX herbal medicine OR TX decoction*<br><br>#3. #1 AND #2
Allied and Complementary Medicine Database via EBSCO	#1. SU Suicide OR SU Self Mutilation OR TX self-harm OR TX self-poisoning OR TX self-injur* OR TX Self Mutilation OR TX attempted suicide OR TX suicid*<br><br>#2. SU Drugs, Chinese Herbal OR SU Medicine, Chinese Traditional OR SU Medicine, Kampo OR SU Medicine, Korean Traditional OR TX Korean medicine OR TX Chinese medicine OR TX Oriental medicine OR TX Kampo medicine OR TX herbal medicine OR TX decoction*<br><br>#3. #1 AND #2
PsycARTICLES via ProQuest	#1. SU (Suicide) OR SU (“Self Mutilation”) OR self-harm OR self-poisoning OR self-injur* OR “Self Mutilation” OR “attempted suicide” OR suicid*<br><br>#2. SU (Drugs, Chinese Herbal) OR SU (Medicine, Chinese Traditional) OR SU (Medicine, Kampo) OR SU (Medicine, Korean Traditional) OR ‘Korean medicine’ OR ‘Chinese medicine’ OR ‘Oriental medicine’ OR ‘Kampo medicine’ OR ‘herbal medicine’ OR ‘decoction*’<br><br>#3. #1 AND #2
China National Knowledge Infrastructure	(SU = '自杀' + '自伤' + 'suicide' + 'self-harm') AND (SU = '中药' + '中医药' + '本草' + '汤' + '丸' + '散' + '方' + '颗粒' + '胶囊' + '自拟')
Wanfang data	SU : ("自杀" or "自伤" or "suicide" or "self-harm") and SU : ("中药" or "中医药" or "本草" or "汤" or "丸" or "散" or "颗粒" or "胶囊") not SU:(针对)
VIP Chinese Science and Technology Periodicals	M = (自杀 or 自伤 or suicide or self-harm) AND M = (中药 or 中医药 or 本草 or 汤 or 丸 or 散 or 颗粒 or 胶囊) NOT M=(针对)
Citation Information by NII	(自殺 OR 自傷 OR suicide OR self-harm) AND (“traditional Chinese medicine” OR “traditional Korean medicine” OR “Traditional oriental medicine” OR “complementary medicine” OR “alternative medicine” OR “Kampo medicine” OR herb OR decoction OR botanic OR 漢方薬 OR 散 OR 汤 OR 丸)
Oriental Medicine Advanced Searching Integrated System	(자살 OR 자해) AND 한약

Koreanstudies Information Service System	(자살 OR 자해) AND 한약
Korea Citation Index	(자살 OR 자해) AND 한약
Research Information Sharing Service	(자살 OR 자해) AND 한약
Korean Medical database	(자살 OR 자해) AND 한약
